# MFCNV: A New Method to Detect Copy Number Variations From Next-Generation Sequencing Data

**DOI:** 10.3389/fgene.2020.00434

**Published:** 2020-05-15

**Authors:** Haiyong Zhao, Tihao Huang, Junqing Li, Guojun Liu, Xiguo Yuan

**Affiliations:** ^1^School of Computer Science and Technology, Liaocheng University, Liaocheng, China; ^2^The School of Computer Science and Technology, Xidian University, Xi’an, China

**Keywords:** copy number variations, next-generation sequencing data, multiple features, neural network, tumor purity

## Abstract

Copy number variation (CNV) is a very important phenomenon in tumor genomes and plays a significant role in tumor genesis. Accurate detection of CNVs has become a routine and necessary procedure for a deep investigation of tumor cells and diagnosis of tumor patients. Next-generation sequencing (NGS) technique has provided a wealth of data for the detection of CNVs at base-pair resolution. However, such task is usually influenced by a number of factors, including GC-content bias, sequencing errors, and correlations among adjacent positions within CNVs. Although many existing methods have dealt with some of these artifacts by designing their own strategies, there is still a lack of comprehensive consideration of all the factors. In this paper, we propose a new method, MFCNV, for an accurate detection of CNVs from NGS data. Compared with existing methods, the characteristics of the proposed method include the following: (1) it makes a full consideration of the intrinsic correlations among adjacent positions in the genome to be analyzed, (2) it calculates read depth, GC-content bias, base quality, and correlation value for each genome bin and combines them as multiple features for the evaluation of genome bins, and (3) it addresses the joint effect among the factors via training a neural network algorithm for the prediction of CNVs. We test the performance of the MFCNV method by using simulation and real sequencing data and make comparisons with several peer methods. The results demonstrate that our method is superior to other methods in terms of sensitivity, precision, and F1-score and can detect many CNVs that other methods have not discovered. MFCNV is expected to be a complementary tool in the analysis of mutations in tumor genomes and can be extended to be applied to the analysis of single-cell sequencing data.

## Introduction

Copy number variations (CNVs) are a type of structural variations accounting for the majority of genomic mutations in human genome. CNVs have been demonstrated to be associated with various complex diseases including cancer, systemic lupus erythematous, Parkinson’s disease, and autoimmune diseases (McCarroll and Altshuler, 2007; Sebat et al., 2007; Stankiewicz and Lupski, 2010). Accurate detection of CNVs from human genome has become a crucial step for a deep understanding of a complex disease and its evolution and has gradually become a regular procedure for designing precision medicine. The recent development of next-generation sequencing (NGS) technique has provided us with an unprecedented opportunity to discover new CNVs. Compared with traditional chromosomal microarray technologies including array comparative genomic hybridization and single nucleotide polymorphism genotyping arrays, NGS has several distinguishable advantages: high-level resolution, high efficiency, and reduction of cost (Schuster, 2008; Ansorge, 2009, 2010). Therefore, it is very attractive and promising for researchers to develop methods for the detection of CNVs and other types of genomic mutations by using NGS data.

Currently, a lot of computational methods have already been proposed to detect CNVs on targeted, whole-exome, or whole-genome sequencing data. These methods could be generally classified into four categories: read depth (RD), paired-end mapping, split-read, and *de novo* assembly (Zhao et al., 2013; Mason-Suares et al., 2016; Yuan et al., 2019a). Since the size of CNVs is typically ranging from 1 kb to several mega bases (Freeman et al., 2006) while the length of the sequencing reads is usually limited to hundreds of bases, the RD-based methods are expected to have the most potential to accurately detect CNVs in a wide range of sizes (Yuan et al., 2019a). One of the most popular RD-based methods is CNVnator (Abyzov et al., 2011), which adopts a mean-shift technique (Comaniciu and Meer, 2002) to partition the observed RD profile into segments with presumably different copy numbers, merges segments with minimal difference in RD by a greedy algorithm, and then makes CNV calls via a *t*-test procedure. The merit of this method is that it can discover broad CNVs and can get a high sensitivity in the analysis of data with high coverage depth. However, when facing with relatively low-coverage-depth data, the false-positive rate of CNVnator is not easy to control due to the influence from artifacts such as GC-content bias and uneven distribution of reads, although the CNVnator method has dealt with the GC bias in a reasonable way. Other popular RD-based methods include ReadDepth (Miller et al., 2011), XCAVATOR (Magi et al., 2017), Wavedec (Cai et al., 2018), seqCNV (Chen et al., 2017), iCopyDAV (Dharanipragada et al., 2018), GROM-RD (Smith et al., 2015), CONDEL (Yuan et al., 2018a), CLImAT (Yu et al., 2014), CNV_IFTV (Yuan et al., 2019b), m-HMM (Wang et al., 2014), DCC (Yuan et al., 2018c), CNV-seq (Xie and Tammi, 2009), and FREEC (Boeva et al., 2012). The characteristics of the existing methods are listed in Table 1. The common steps behind most of these methods include: (1) read processing, e.g., filtering low-quality bases, (2) read alignment to the reference genome, (3) dividing the genome into non-overlapping bins of equal sizes and calculating the RD for each bin, (4) correcting GC-content bias across the RD profile, and (5) calling CNVs through establishing computational models on the RD profile. The primary difference among these methods lies in their insights into the RD profile from different perspectives. For example, Wavedec explores the shared and individual CNV patterns with different characteristics via wavelet transform on the RD profile, m-HMM models the RD profile as a series of hidden states, and CNV_IFTV models the RD profile as a forest of trees. Such methods exhibit their own advantages in the application to synthetic and real sequencing datasets. However, many factors related with CNVs and the interactions between them have still not been explored fully by the existing methods.

**TABLE 1:** The characteristics of the existing methods.

**Methods**	**Signals**	**Analysis samples**	**Data input**	**Language and interface**
ReadDepth	Read depth	Single	BED	R, CLI
XCAVATOR	Read count	Multiple, matched, single	Read count	Perl, bash, R, Fortran, CLI
Wavedec	Read depth	Multiple, single	Array, aCGH, read depth	–
seqCNV	Read count	Matched	BAM and BED	Perl, CLI
iCopyDAV	Read depth	Matched	BAM and BED	C+++, R, CLI
GROM-RD	Read depth	Single	BAM and BED	C, CLI
CONDEL	Read depth	Single	Read depth	C++, Perl, CLI
CNV IFTV	Read depth	Single	Read depth	Python, CLI
m-HMM	Read count	Matched	Positions and read count	R, CLI
DCC	Read depth	Multiple	Read depth	C++, Perl, CLI
CNV-seq	Read depth	Single	BED	R, Perl, CLI
FREEC	Read depth and GC content	Single, matched	BAM, SAM	C++, CLI

*Single, single sample analysis with normal-matched samples; matched, tumor-normal matched samples; multiple, multiple sample analysis; CLI, command line.*

Besides the values of RDs, the intrinsic correlations among adjacent genomic positions are closely associated with CNVs (Yuan et al., 2012b, 2018a). An independent analysis of RDs tends to result in conservativeness in the calling of CNVs. Although some existing methods have implicitly addressed the effect of correlations, such as CNVnator (Abyzov et al., 2011) and CONDEL (Yuan et al., 2018a), which improve the efficiency of statistics on RDs via partitioning the genome into independent segments and combining the correlations into a statistic design, respectively, the interaction between RDs and correlations is still ignored. The neglect of such interaction may limit their generalization abilities in detecting CNVs under various scenarios of sequencing data (e.g., low coverage depth and low tumor purity). In addition, other factors, such as GC-content bias and base quality, have also been dealt with separately. Generally, a separate analysis of these factors is just suitable to some particular scenarios. For example, if the sequencing coverage depth or tumor purity is at a low level, then the proportion of absolute RD signals in the observed signals may not be dominant. In this case, an inappropriate correction of GC-content bias and filtering of low-quality bases with a cutoff will transfer unexpected deviations to the RD profiles so that the analysis of CNVs is influenced accordingly. Therefore, a reliable and feasible strategy is to make a full consideration about the CNV-related factors by addressing the interactions among them.

With the concerns above, in this paper, we propose a new method, called MFCNV (detection of CNVs based on multiple features), for an accurate detection of CNVs from NGS data. The primary principle of MFCNV is that it incorporates four factors (RD, GC-content, correlations among adjacent genomic positions, and base quality) into the analysis of genome bins and makes a joint effect among them via training a neural network algorithm for the prediction of CNVs. Compared with existing methods, it has considered the major factors related with CNVs, especially for the intrinsic correlations among adjacent genomic positions that very few methods have been concerned of. Moreover, MFCNV addresses the interactions among the incorporated factors rather than marginal effects. To demonstrate the performance of MFCNV, we carry out experiments on simulation and real sequencing samples and compare it with several peer methods. The comparative results indicate the merits of MFCNV over the other methods. MFCNV can be expected to be a routine approach for the detection of new CNVs from NGS data in real applications and can be extended to analyze single-cell sequencing data.

The remainder of this paper is organized as follows: The workflow of MFCNV and the related principles are described firstly, and then simulation studies are designed to evaluate the performance of the proposed method and its peer methods as well as validations by applying it to real sequencing samples. Finally, the proposed method is discussed and an outline of future work for improvements is illustrated.

## Materials and Methods

### Workflow of MFCNV

The workflow chart of the MFCNV method is illustrated inFigure 1. It starts from an initial input of a reference genome and a sequencing sample to a preprocessing of the input data and then carries out four primary steps to realize the prediction of CNVs. Here the reference genome can be chosen from the latest version of references such as GRCh38, and the sequencing sample to be analyzed can be obtained from tumor samples without normal matched samples. The four primary steps include: (1) definition of factors related to CNVs, where four types of factors are calculated and their values are normalized, (2) construction of a neural network based on the factors, where a back-propagation (BP) neural network algorithm is selected, (3) training of the neural network, where labeled CNVs could be sampled from both synthetic and real sequencing datasets, and (4) prediction of CNVs and declaration of gains or losses, where the CNV state for each genome bin is predicted based on the trained neural network algorithm as well as the type of CNVs (gain or loss). The codes of the MFCNV method are freely available at the website https://github.com/BDanalysis/mfcnv. In the following text, we make a detailed description of the principle for each of the four steps.

**FIGURE 1:**
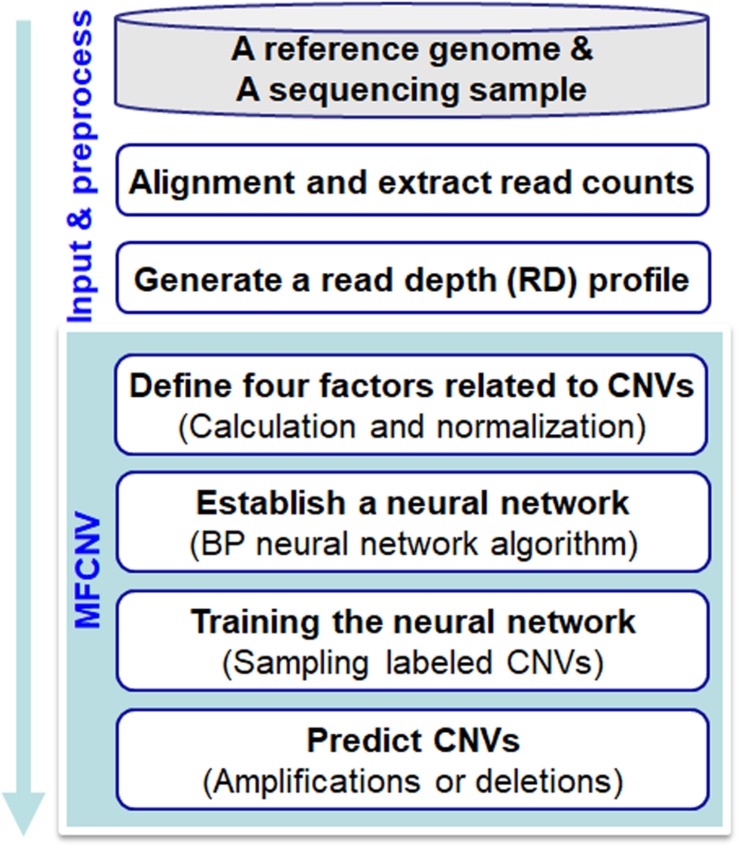
Workflow of the MFCNV method for the detection of copy number variations from next-generation sequencing data.

### Data Input and Preprocess

The input two files (i.e., a reference file formatted in fasta and a sequencing sample file formatted in fastq) are handled by adopting one of the classic alignment approaches BWA (Li and Durbin, 2009). This will result in an alignment file formatted in BAM. A read count (RC) profile can be obtained from this BAM file by using the SAMtools software (Li et al., 2009). The template of the RC profile is the reference genome, where a large majority of the positions have been determined with the regular bases (“A,” “T,” “C,” and “G”), while a small fraction of them have not been determined (Yuan et al., 2019a). The undetermined positions are usually filled with letter “N” so that no sequencing reads could be matched to these areas. To obtain a complete and reasonable RC profile, we discard the “N” areas in the reference template. This is similar to our previous work (Yuan et al., 2019a). However, this will inevitably lead to a situation: the observed mean RC across the whole genome is smaller than the expected coverage depth since a set of sequencing reads that originated from “N” areas are unmapped. This implies that taking the mean RC as a baseline of normal copy number may not be reasonable in the calling of CNVs. Therefore, in our work, we do not rely on such baseline but train a predictor for the declaration of CNVs. This will be described in detail in the following text.

For the calculation of the RD profile, we divide the extracted RC profile into non-overlapping and continuous genome bins of equal sizes. The mean RC within each genome bin is taken as the RD value. The bin size can be set to 1,000 or 2,000 bp or other suitable values according to the users (Yuan et al., 2018a, c). Generally, if we focus on detecting broad CNVs, a large bin size should be preferred, while if we focus on detecting focal CNVs, a small bin size should be preferred. It is a fact that the larger the bin size, the larger the boundary deviation from the ground truth might be. Another factor that affects the setting of bin size is the sequencing coverage depth. If the coverage depth is at an extremely low level (e.g., less than 2×), a small bin size tends to produce a high false-positive rate. This is because many bins may present zero RD values, which cannot be discriminated from real homogeneous deletions. If the coverage depth is at a moderate or high level, a relatively small bin size could be chosen, which can narrow the deviations between the detected CNVs and the ground truths without losing the sensitivity. Therefore, we take the bin size as a flexible parameter in the design of the MFCNV software rather than fix its value. For convenience, we denote the template of bins and the RD profile by using Eqs (1) and (2), respectively.

(1) $$B=\left\{b_{1},b_{2},b_{3},\ldots ,b_{n}\right\},$$

(2) $$R=\left\{r_{1},r_{2},r_{3},\ldots ,r_{n}\right\},$$

where *n* represents the total number of bins in the whole genome to be analyzed, *b*_i_ represents the *i*-th bin, and *r*_i_ represents the RD value of the *i*-th bin. Our purpose is to determine whether each *b*_i_ is abnormal or normal and then further determine if it is an amplification or a deletion event.

### Definition of Factors Related to CNVs

Based on the template of *B*, the RD profile *R* can be used to establish statistical or computational models for testing each bin in *B*. However, the RD profile is usually influenced by some artifacts such as GC-content bias, sequencing errors, and even uneven distributed sequencing reads. This may lead to the observed *R* which may not be a reasonable reflection of copy numbers across the whole genome. If the statistical or computational models are established directly on the observed *R*, the performance of calling CNVs is difficult to guarantee. Although many existing methods have made preprocess procedures to deal with some artifacts, the effect may be suitable to some particular situations according to our analysis in the previous text. In this work, we do not make a correction to the GC-content bias beforehand but take the GC-content as a factor for the detection of CNVs. At the same time, we do not deliberately discard the bases with qualities below a predefined-cutoff but also take the quality as a factor. Except for the factors that can influence the RD profile, some characteristics associated with structural variation itself should be taken into consideration. The intrinsic correlation among adjacent genome positions is one of the most important characteristics. Accordingly, we have obtained a total of four factors including RD for the prediction of CNVs. Since the RD profile is definitely given in Eq. (2), below we focus on the definition of the other three factors, including their calculations and normalizations.

In order to maintain the consistency of the representation format, we denote the GC-content, base quality, and correlation by using Eqs (3–5), respectively, where *n* represents the total number of bins in the whole genome to be analyzed, *g*_i_, *q*_i_, and *c*_i_ represents the fraction of GC-content, average quality, and correlation of the *i*-th bin, respectively. Accordingly, each bin *b*_i_ can be represented by using a quadruple, *b*_i_ = (*r*_i_, *g*_i_, *q*_i_, *c*_i_). Now our purpose is to determine whether *b*_i_ is a CNV or not according to the quadruple.

(3) $$G=\left\{g_{1},g_{2},g_{3},\ldots ,g_{n}\right\},$$

(4) $$Q=\left\{q_{1},q_{2},q_{3},\ldots ,q_{n}\right\},$$

(5) $$C=\left\{c_{1},c_{2},c_{3},\ldots ,c_{n}\right\}.$$

As far as calculations of the factors are concerned, *g*_i_ is defined as the fraction of “G” and “C” within the *i*-th bin and its value is ranging from 0 to 1, and *q*_i_ is defined as the average quality of the mapped reads within the *i*-th bin. As for *c*_i_, it is not easy to calculate based on a single sample, instead we extract a value that can reflect the smoothness of the *i*-th bin with its surrounding area. This value is obtained by using Eq. (6).

(6) $$c_{i}=\left|r_{i}-\frac{1}{2\,w}\,\sum_{i-w,j\neq i}^{i+w}r_{j}\right|,$$

where *w* denotes the number of left adjacent and right adjacent bins for the *i*-th bin. The meaning of Eq. (6) can be explained as such that the RD difference between the *i*-th bin and its adjacent bins should be small if they are within the same copy number segment; otherwise, the difference should be relatively large. The fundamental concept underlying such explanation is that copy numbers are structural and intrinsic-correlated.

By far, we could achieve the values for all the elements in *b*_i_. Since each element may have different ranges of values, for example, the value of *q*_i_ is usually dozens while the value of *g*_i_ is limited to 1, it is necessary and meaningful to make normalizations for the values so that they are at the same level. Such procedure will bring a balanced adjustment effect on various factors. The normalization strategy for GC content can be expressed as Eq. (7), and it is similar for other factors. Here to maintain the values of the observed RDs, we use a weight to make a scaling of the normalized values for some factors.

(7) $$G=\frac{G}{\max\left\{g_{1},g_{2},g_{3},\ldots ,g_{n}\right\}},$$

### Establishment of a Neural Network

**FIGURE 2:**
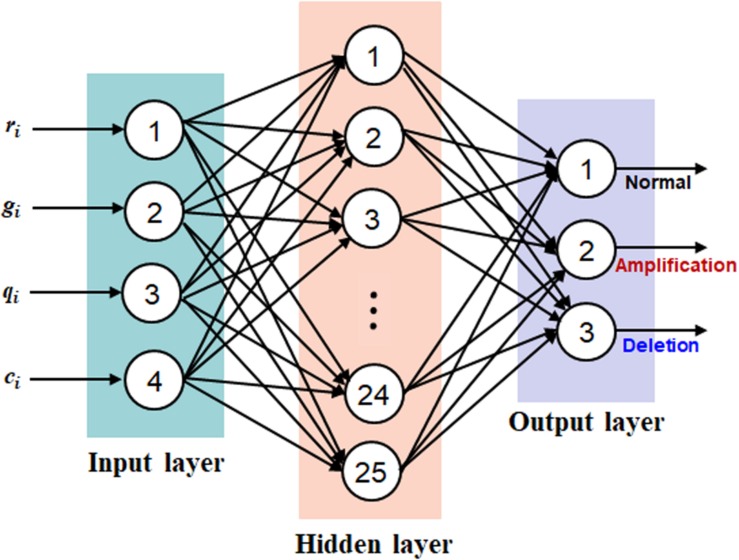
The topology of the back-propagation neural network, which includes three layers: input layer with four neurons, hidden layer with 25 neurons, and output layer with three neurons.

Based on the representation of each bin with a quadruple, *b*_i_ = (*r*_i_, *g*_i_, *q*_i_, *c*_i_), we establish a neural network on the four factors for the prediction of CNVs. Such approach implicitly addresses the interaction between the four factors, producing a joint effect, rather than a separate analysis of each factor with a marginal effect. We take the four factors as four types of features to establish a neural network, which can model the potential interaction between features and adapt to changing input to generate the best possible result. Here we choose the classic BP algorithm (Rumelhart et al., 1986) as the basic architecture of the neural network. This type of neural network is simple in topology and has a nonlinear mapping ability and thus has been commonly applied in a wide range of fields such as pattern recognition, image processing, and natural language processing (Huang et al., 2005; Chen, 2018; Xue and Cui, 2019; Zhao et al., 2019). Generally, the BP neural network is composed of three categories of layers including input, hidden, and output layers, where users can set multiple hidden layers according to their requirements. In our work, we set three layers in the BP neural network and each of them is corresponding to each type of layer. The topology of the BP neural network is illustrated in Figure 2, where the input layer is composed of four neurons, the hidden layer is composed of 25 neurons, and the output layer is composed of three neurons. The input layer is responsible for the input of the four types of features, and the output layer is responsible for the output of normal, amplification, and deletion copy numbers. In terms of the hidden layer, the number of neurons is determined according to our experience on extensive testing of the algorithm. For the hidden and output layers, we consistently utilize the Sigmoid function as the activation function, which is expressed by Eq. (8).

(8) $$f\,\left(w^{T}\,x+b\right)=\frac{1}{1+e^{-\left(w^{T}\,x+b\right)}},$$

where *w* represents the weights between any two layers, *b* represents the corresponding biases, and *x* represents the neuron input vector from its previous layer. For a more detailed description about the theorem of the BP neural network, refer to (Rumelhart et al., 1986; Liu et al., 2019).

### Training of the Neural Network

A reasonable training of the BP neural network algorithm is a crucial step before putting it into practical applications. One of the key parameters to the algorithm is the learning rate, which is strongly associated with the learning speed and convergence. Finding an appropriate learning rate can guarantee a fast and robust convergence (Jing, 2012). According to our experience on extensive testing of the algorithm, we choose an appropriate value of 0.1 as the learning rate. Such value is moderate (Rumelhart et al., 1986; Chen et al., 2004) and is the default value in the BP neural network algorithm.

In addition to the parameter setting, another important point to influence the training effect of the algorithm is the collection of training datasets. Generally, the BP neural network algorithm could be trained by using both simulation and real sequencing datasets. Since the input to the algorithm is a quadruple, we represent the training dataset by incorporating a column of labels, *T*_m__×__5_ = (*r, g, q, c, L*), where *m* denotes the number of bins to be used for training, *r, g, q*, and *c* are column vectors that represent the values of the four types of features, and *L* is a column vector that represents the labels of the bins. Now the task is to collect the set of bins labeled with normal, amplification, and deletion. Generally, simulation sequencing samples could provide definite ground truth CNVs from which the bins with different labels could be smoothly extracted. Meanwhile, the ground truth CNVs in real sequencing samples are not easy to obtain since it is difficult for researchers to label regions absolutely correctly. Therefore, collecting labeled bins from simulation datasets might be reasonable and reliable. It should be noticed that, in the real world, genomes sequenced from tumor tissues are usually contaminated with a fraction of normal cells. This means that the bins in the samples with different levels of tumor purity may display different feature values. Therefore, to facilitate the generalization performance of the trained algorithm, it is suggested to collect bins from samples with various levels of tumor purity, even with various sequencing coverage depths. In addition, the training dataset should be dynamic, i.e., it can be updated by adding new labeled bins or removing low-quality bins along with the continuous application of the trained algorithm. Such dynamic updating to the training dataset can help to improve the ability of the algorithm. In this work, we use various individuals sampled from different sequencing coverage depths and different tumor purity levels, and in each individual we extracted a great number of bins represented by quadruples with labels to train the algorithm. In the training datasets, no chromatin elements and DNase hypersensitive sites were included.

### Prediction of CNVs

With the trained BP neural network algorithm, we can perform the prediction of CNVs on testing datasets. For each bin, the output of the algorithm includes three values mapped from the Sigmoid function. These values are ranging from 0 to 1, representing the probabilities of one bin belonging to normal, amplification, or deletion state, respectively. We determine the state of one bin according to the largest probability. For example, if the probability of amplification is larger than the other two, then the bin is declared as amplification. After all the bins across the whole genome have been declared, the adjacent bins with the same copy number state can be merged into a segment, and then the segments will be finally output with copy number states (normal, amplification, or deletion).

## Results

### Simulation Studies

The performance evaluation is crucial to decide whether the proposed method is valid or not. Simulation studies are usually regarded as an appropriate and feasible way to evaluate performance for existing and newly developed methods (Yuan et al., 2012a, 2017, 2018b). This is because the ground truth underlying the simulation samples could be used for an exact calculation of true-positive and false-positive rates. In addition, simulation studies can assist us to seek for an appropriate parameter setting for the algorithm. To mirror this, we carry out simulation experiments to test our proposed method and to make comparisons with several peer methods [CNAnator (Abyzov et al., 2011), FREEC (Boeva et al., 2012), GROM_RD (Smith et al., 2015), and iCopyDAV (Dharanipragada et al., 2018)]. To ensure a fair comparison between our method and the four peer methods, we use the default parameter settings of the peer methods in the running of their algorithms. The command lines of executing these algorithms can be referred to the manuals of the corresponding software packages.

**FIGURE 3:**
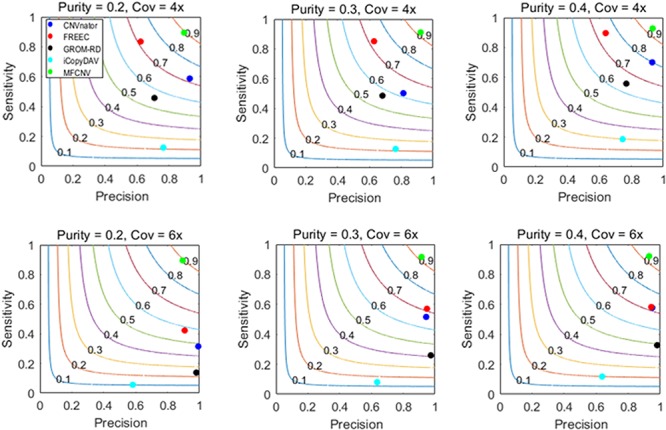
Performance comparisons between our proposed method and the four peer methods in terms of sensitivity, precision, and F1-score (colored curves) on simulation datasets.

The design of the simulation experiments is described as follows: we adopt our previously developed simulation tool IntSIM (Yuan et al., 2017) to produce various datasets with varying tumor purity from 0.2 to 0.4 and varying sequencing coverage depth from 4× to 6× (Yuan et al., 2019b). In each simulation configuration, 50 replicated samples are generated for a sufficient test of our proposed method and the peer methods. In each replicated sample, 14 CNVs have been simulated with a size ranging from 10,000 to 500,000 bp. We implement the five methods on these datasets. To guarantee a fair comparison, we always use the default parameter settings for the methods to be compared. The comparative results are illustrated in Figure 3, where sensitivity, precision, and F1-score (colored curves) are presented. Here the presented sensitivity is the averaged value over the 50 replicated samples as well as the presented precision and F1-score. The sensitivity is calculated as the number of correctly detected CNVs (true-positive) divided by the total number of ground truth CNVs, and the precision is calculated as the number of correctly detection CNVs divided by the total number of calls. For MFCNV, true-positives are counted based on the unit of bins, i.e., if a bin is correctly labeled, it is declared as a true-positive. For the four peer methods, true-positives are counted based on the unit of the markers since their output CNVs is a list of segments with different lengths.

From Figure 3, we could observe that the performances of all the methods are improving along with the increase of tumor purity. For example, the F1-score of the CNVnator method is around 0.7 at a tumor purity of 0.2 and a coverage depth of 4×, while it gets to 0.8 at a tumor purity of 0.4 and a coverage depth of 4×. For precision, the CNVnator ranks first in two of six cases, GROM_RD ranks first in two of the other four cases, and MFCNV ranks first in the rest of the two cases. For sensitivity, MFCNV is superior in most of the six cases, followed by FREEC, CNVnator, GROM_RD, and then iCopyDAV. As far as F1-score is concerned, MFCNV obtains the largest values in all the six cases, followed by CNVnator or FREEC, and then the other methods. This means that MFCNV displays the best trade-off between sensitivity and precision in the detection of CNVs under these simulation experiments.

The advantage of MFCNV over the four peer methods can be explained from two primary perspectives. On the one hand, MFCNV addresses the joint effect among multiple factors associated with CNVs by establishing a neural network algorithm, while the other methods emphasized on the marginal effects of the factors. On the other hand, we train the MFCNV algorithm by selecting a great number of bins from different samples with various tumor purities and sequencing coverage depths. Such strategy can improve the adaptability of the method in facing different scenarios of datasets.

### Real Data Applications

**FIGURE 4:**
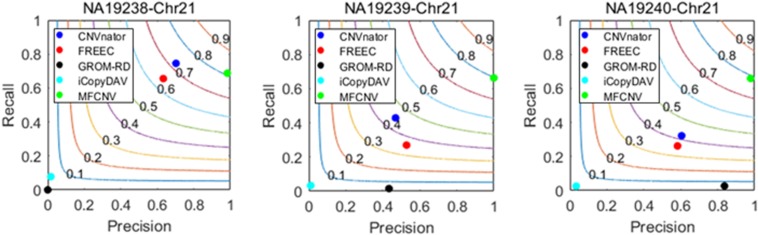
Performance comparisons between our proposed method and the four peer methods in terms of recall, precision, and F1-score (colored curves) on real samples.

To validate the usefulness of our proposed method, we apply it to analyze three real samples, NA19238, NA19239, and NA19240, which are obtained from the CEU family trio and can be accessed at the 1000 Genomes Project^1^. We perform the MFCNV method and the four peer methods on chromosome 21 for each of the samples. In these experiments, MFCNV have detected 233, 181, and 183 CNVs for the three samples, respectively. The total number of CNVs detected by MFCNV is larger than that detected by any other method (shown in Table 2). For these samples, the database of genomic variants (DGV)^2^ has provided the confirmed CNVs, which can be used to quantify the performance of our method and of the other four methods. The comparative results with respect to recall, precision, and F1-score are depicted in Figure 4. Here the calculation of these evaluation indices are based on the bin unit, i.e., a true-positive is counted when one bin in the ground truth CNVs is declared as CNV.

**TABLE 2:** Comparison of the number of detected copy number variations between MFCNV and the four peer methods on real samples.

**Sample**	**CNVnator**	**FREEC**	**GROM-RD**	**iCopyDAV**	**MFCNV**
NA19238	252	222	0	26	233
NA19239	145	91	5	11	181
NA19240	109	88	9	8	183

From Figure 4, we could observe that CNVnator has got the highest recall for the sample NA19238, while MFCNV has got the highest recalls for the remaining two samples, followed by FRECC and the other methods. As for precision, MFCNV is superior to the other methods for all the three samples, followed by CNVnator, FREEC, or GROM-RD. In terms of F1-score, MFCNV ranks first and has got a value of around 0.8 for all the three samples. Such comparative results demonstrate that our proposed method has the best trade-off between recall and precision over the other methods. This implies that MFCNV is reliable in the applications to real sequencing samples, although we cannot guarantee that the ground truth in the DGV database is absolutely correct and complete.

## Discussion

Accurate detection of CNVs is a crucial step for a comprehensive analysis of genomic mutations in the study of genome evaluation and human complex diseases. In this paper, we propose a new computational method, MFCNV, for the detection of CNVs from NGS data. The major principle of the proposed method is that it integrates multiple CNV-related features and trains a neural network algorithm to predict CNVs. It is different from most of the existing methods, which separately analyze the factors associated with copy numbers and just focus on the significance of RD signatures on CNVs, while our method addresses the joint effect between different factors. Compared with the strategies of existing methods, the advantages of MFCNV include the following: (1) it does not require a GC-content bias correction procedure so that it can avoid the errors transferred from the procedure to the downstream analysis, (2) it extracts four effective features that are closely associated with CNVs and genome structures and utilizes a nonlinear model (i.e., neural network) to explore the interaction between them; such interaction may be more powerful than the marginal effect in the separate analysis of factors, and (3) it trains the neural network algorithm by using datasets with various levels of tumor purities and sequencing coverage depths; this will improve the adaptability and generalization performance of the algorithm.

Generally, each of the four selected features may have its own marginal effect and may pose different significance to the CNV prediction. Here, we integrate the four features and train a neural network algorithm to predict CNVs. In the training of the neural network, the connection weights can be learned and can reflect the marginal effects of the corresponding features. Meanwhile, their integration can produce a joint effect for the prediction of CNVs. Since different features may have different ranges of values, we make normalization for the values before inputting them to the neural network. The normalization is conducted to scale the feature values at the same level so that the values of the different features can be balanced.

For the performance evaluation, we first simulate a large number of synthetic datasets with various configurations and test MFCNV in terms of sensitivity, precision, and F1-score. The experimental results indicate that MFCNV can get a higher performance than the four peer methods. Furthermore, we carry out experiments on three real samples and utilize the confirmed CNVs in the DGV database to quantify the performance of MFCNV and the peer methods. The results also demonstrate the merits of MFCNV. Therefore, we could conclude that MFCNV is a valid and reliable method in the detection of CNVs by using NGS data and is expected to become a complementary and regular tool in the field of genomic mutation analysis.

For the future work, we intend to make improvements to the current version of MFCNV from the following aspects: In the first place, the contamination of normal genomes in the sequenced genomes is an important factor to influence the power of CNV detection (Yu et al., 2011; Yuan et al., 2017). Thus, it is meaningful to integrate the module of tumor purity prediction into the pipeline of CNV detection. In the second place, single-cell sequencing technique has provided new opportunities to analyze genomic mutations at individual cells. The genome bins including aberration and normal states from such sequencing data can be collected to train the algorithm of MFCNV, which can then be extended to detect CNVs from single-cell sequencing data. In the last place but not the least, an alternative architecture of the neural network can be designed, i.e., the output layer of the current neural network with three neurons can be placed with only one neuron. This neuron is responsible for determining whether one genome bin is abnormal or normal. If it is abnormal, we further compare its RD value to the mode of RDs to decide whether it is amplification or deletion. Such decrease in the number of neurons in the output layer may improve the efficiency of the algorithm.

## Data Availability Statement

Publicly available datasets were analyzed in this study: NA19238, NA19239, and NA19240, which are obtained from the CEU family trio and can be accessed at the 1000 Genomes Project (http://www.internationalgenome.org/).

## Author Contributions

HZ and TH participated in the design of algorithms and experiments. JL and HZ participated in the design of the whole framework of detecting CNVs. XY directed the whole work. GL participated in the analysis of the performance of the proposed method. JL and XY conceived the study and helped in editing the manuscript. All authors read the final manuscript and agreed on its submission.

## Conflict of Interest

The authors declare that the research was conducted in the absence of any commercial or financial relationships that could be construed as a potential conflict of interest.

## Footnotes

1 http://www.internationalgenome.org/

2 http://dgv.tcag.ca/

## References

[B1] Abyzov A. Urban A. E. Snyder M. Gerstein M. (2011). CNVnator: an approach to discover, genotype, and characterize typical and atypical CNVs from family and population genome sequencing. *Genome Res.* 21 974–984. 10.1101/gr.114876.110 21324876 PMC3106330

[B2] Ansorge W. J. (2009). Next-generation DNA sequencing techniques. *New Biotechnol.* 25 195–203. 10.1016/j.nbt.2008.12.009 19429539

[B3] Ansorge W. J. (2010). Next generation DNA sequencing techniques and applications. *New Biotechnol.* 27 S3–S3. 10.1016/j.nbt.2008.12.009 19429539

[B4] Boeva V. Popova T. Bleakley K. Chiche P. Cappo J. Schleiermacher G. (2012). Control-FREEC: a tool for assessing copy number and allelic content using next-generation sequencing data. *Bioinformatics* 28 423–425. 10.1093/bioinformatics/btr670 22155870 PMC3268243

[B5] Cai H. Chen P. Chen J. Cai J. Song Y. Han G. (2018). WaveDec: a wavelet approach to identify both shared and individual patterns of copy-number variations. *IEEE Trans. Biomed. Eng.* 65 353–364. 10.1109/TBME.2017.2769677 29346103

[B6] Chen Y. Zhao L. Wang Y. Cao M. Gelowani V. Xu M. C. (2017). SeqCNV: a novel method for identification of copy number variations in targeted next-generation sequencing data. *BMC Bioinformatics* 18:147. 10.1186/s12859-017-1566-3 28253855 PMC5335817

[B7] Chen Y. G. (2018). Prediction algorithm of PM2.5 mass concentration based on adaptive BP neural network. *Computing* 100 825–838.

[B8] Chen Y. J. Huang T. C. Hwang R. C. (2004). An effective learning of neural network by using RFBP learning algorithm. *Inform. Sci.* 167 77–86.

[B9] Comaniciu D. Meer P. (2002). Mean shift: a robust approach toward feature space analysis. *IEEE Trans. Pattern Anal. Mach. Intell.* 24 603–619. 21428083

[B10] Dharanipragada P. Vogeti S. Parekh N. (2018). iCopyDAV: integrated platform for copy number variations-detection, annotation and visualization. *PLoS One* 13:e0195334. 10.1371/journal.pone.0195334 29621297 PMC5886540

[B11] Freeman J. L. Perry G. H. Feuk L. Redon R. McCarroll S. A. Altshuler D. M. (2006). Copy number variation: new insights in genome diversity. *Genome Res.* 16 949–961. 10.1101/gr.3677206 16809666

[B12] Huang X. M. Tang Z. Sun C. X. (2005). “Study of BP neural network and its application in lung cancer intelligent diagnosis,” in *Proceedings of the 2nd International Symposium on Neural Networks: Advances in Neural Network*, Vol. 3498 Chongqing, 774–779.

[B13] Jing X. (2012). Robust adaptive learning of feedforward neural networks via LMI optimizations. *Neural Netw.* 31 33–45. 10.1016/j.neunet.2012.03.003 22459273

[B14] Li H. Durbin R. (2009). Fast and accurate short read alignment with Burrows-Wheeler transform. *Bioinformatics* 25 1754–1760. 10.1093/bioinformatics/btp324 19451168 PMC2705234

[B15] Li H. Handsaker B. Wysoker A. Fennell T. Ruan J. Homer N. (2009). The sequence Alignment/Map format and SAMtools. *Bioinformatics* 25 2078–2079. 10.1093/bioinformatics/btp352 19505943 PMC2723002

[B16] Liu Y. Zhao Q. Yao W. Ma X. Yao Y. Liu L. (2019). Short-term rainfall forecast model based on the improved BP-NN algorithm. *Sci. Rep.* 9 19751. 10.1038/s41598-019-56452-5 31875049 PMC6930286

[B17] Magi A. Pippucci T. Sidore C. (2017). XCAVATOR: accurate detection and genotyping of copy number variants from second and third generation whole-genome sequencing experiments. *BMC Genomics* 18:747. 10.1186/s12864-017-4137-0 28934930 PMC5609061

[B18] Mason-Suares H. Landry L. Lebo M. S. (2016). Detecting copy number variation via next generation technology. *Curr. Genet. Med. Rep.* 4 74–85.

[B19] McCarroll S. A. Altshuler D. M. (2007). Copy-number variation and association studies of human disease. *Nat. Genet.* 39 S37–S42. 10.1038/ng2080 17597780

[B20] Miller C. A. Hampton O. Coarfa C. Milosavljevic A. (2011). ReadDepth: a parallel R package for detecting copy number alterations from short sequencing reads. *PLoS One* 6:e16327. 10.1371/journal.pone.0016327 21305028 PMC3031566

[B21] Rumelhart D. E. Hinton G. H. Rj W. (1986). *Learning Internal Representations by Error Propagation.* Cambridge, MA: MIT Press, 399–421.

[B22] Schuster S. C. (2008). Next-generation sequencing transforms today’s biology. *Nat. Methods* 5 16–18. 10.1038/nmeth1156 18165802

[B23] Sebat J. Lakshmi B. Malhotra D. Troge J. Lese-Martin C. Walsh T. (2007). Strong association of de novo copy number mutations with autism. *Science* 316 445–449. 10.1126/science.1138659 17363630 PMC2993504

[B24] Smith S. D. Kawash J. K. Grigoriev A. (2015). GROM-RD: resolving genomic biases to improve read depth detection of copy number variants. *PeerJ* 3:e836. 10.7717/peerj.836 25802807 PMC4369336

[B25] Stankiewicz P. Lupski J. R. (2010). Structural variation in the human genome and its role in disease. *Annu. Rev. Med.* 61 437–455. 10.1146/annurev-med-100708-204735 20059347

[B26] Wang H. Nettleton D. Ying K. (2014). Copy number variation detection using next generation sequencing read counts. *BMC Bioinformatics* 15:109. 10.1186/1471-2105-15-109 24731174 PMC4021345

[B27] Xie C. Tammi M. T. (2009). CNV-seq, a new method to detect copy number variation using high-throughput sequencing. *BMC Bioinformatics* 10:80. 10.1186/1471-2105-10-80 19267900 PMC2667514

[B28] Xue H. Z. Cui H. W. (2019). Research on image restoration algorithms based on BP neural network. *J. Vis. Commun. Image R.* 59 204–209.

[B29] Yu G. Zhang B. Bova G. S. Xu J. Shih Ie M. Wang Y. (2011). BACOM: in silico detection of genomic deletion types and correction of normal cell contamination in copy number data. *Bioinformatics* 27 1473–1480. 10.1093/bioinformatics/btr183 21498400 PMC3102226

[B30] Yu Z. Liu Y. Shen Y. Wang M. Li A. (2014). CLImAT: accurate detection of copy number alteration and loss of heterozygosity in impure and aneuploid tumor samples using whole-genome sequencing data. *Bioinformatics* 30 2576–2583. 10.1093/bioinformatics/btu346 24845652 PMC4155249

[B31] Yuan X. Bai J. Zhang J. Yang L. Duan J. Li Y. (2018a). CONDEL: detecting copy number variation and genotyping deletion zygosity from single tumor samples using sequence data. *IEEE/ACM Trans. Comput. Biol. Bioinform.* 10.1109/TCBB.2018.2883333 30489272

[B32] Yuan X. Gao M. Bai J. Duan J. (2018b). SVSR: a program to simulate structural variations and generate sequencing reads for multiple platforms. *IEEE/ACM Trans. Comput. Biol. Bioinform.* 10.1109/TCBB.2018.2876527 30334804

[B33] Yuan X. Li J. Bai J. Xi J. (2019a). A local outlier factor-based detection of copy number variations from NGS data. *IEEE/ACM Trans. Comput. Biol. Bioinform.* 10.1109/TCBB.2019.2961886 31880558

[B34] Yuan X. Miller D. J. Zhang J. Herrington D. Wang Y. (2012a). An overview of population genetic data simulation. *J. Comput. Biol.* 19 42–54. 10.1089/cmb.2010.0188 22149682 PMC3244809

[B35] Yuan X. Yu G. Hou X. Shih Ie M. Clarke R. Zhang J. (2012b). Genome-wide identification of significant aberrations in cancer genome. *BMC Genomics* 13:342. 10.1186/1471-2164-13-342 22839576 PMC3428679

[B36] Yuan X. Yu J. Xi J. Yang L. Shang J. Li Z. (2019b). CNV_IFTV: an isolation forest and total variation-based detection of CNVs from short-read sequencing data. *IEEE/ACM Trans. Comput. Biol. Bioinform.* 10.1109/TCBB.2019.2920889 31180897

[B37] Yuan X. Zhang J. Yang L. (2017). IntSIM: an integrated simulator of next-generation sequencing data. *IEEE Trans. Biomed. Eng.* 64 441–451. 10.1109/TBME.2016.2560939 27164567

[B38] Yuan X. Zhang J. Yang L. Bai J. Fan P. (2018c). Detection of significant copy number variations from multiple samples in next-generation sequencing data. *IEEE Trans. Nanobiosci.* 17 12–20. 10.1109/TNB.2017.2783910 29570071

[B39] Zhao G. D. Zhang Y. W. Shi Y. Q. Lan H. Y. Yang Q. (2019). The application of BP neural networks to analysis the national vulnerability. *Comput. Mater. Con.* 58 421–436.

[B40] Zhao M. Wang Q. G. Wang Q. Jia P. L. Zhao Z. M. (2013). Computational tools for copy number variation (CNV) detection using next-generation sequencing data: features and perspectives. *BMC Bioinformatics* 14:S1. 10.1186/1471-2105-14-S11-S1 24564169 PMC3846878

